# Prognostic Value of Red Blood Cell Distribution Width in Predicting Acute Kidney Injury After Cardiac Surgery: A Retrospective Cohort Study

**DOI:** 10.3390/jcm15062403

**Published:** 2026-03-21

**Authors:** Daniele Marianello, Antonella Puddu, Cesare Biuzzi, Alberto Fogagnolo, Savino Spadaro, Lucrezia Galasso, Alessandra Cartocci, Francesco Lorenzo De Matteis, Sandro Sponga, Fabio Silvio Taccone, Sabino Scolletta, Federico Franchi

**Affiliations:** 1 Cardiothoracic and Vascular Anesthesia and Intensive Care Unit, Department of Medical Science, Surgery and Neurosciences, University Hospital of Siena, 53100 Siena, Italy; 2 Anesthesia and Intensive Care Unit, Department of Medicine, Surgery and Neurosciences, University Hospital of Siena, 53100 Siena, Italy; 3 Department of Translational Medicine for Romagna, University of Ferrara, 44121 Ferrara, Italy; 4 Clinical Pathology Unit, Innovation, Experimentation and Clinical and Translational Research Department, University Hospital of Siena, 53100 Siena, Italy; 5 Department of Molecular and Developmental Medicine, University of Siena, 53100 Siena, Italy; 6 Division of Cardiac Surgery, Department of Cardiothoracic and Vascular Disease, University of Siena, 53100 Siena, Italy; 7 Department of Intensive Care, Hôpital Universitaire de Bruxelles (HUB), Université Libre de Bruxelles (ULB), 1070 Brussels, Belgium

**Keywords:** red blood cell distribution width, acute kidney injury, cardiac surgery, cardiopulmonary bypass, biomarkers

## Abstract

**Background/Objectives**: Acute kidney injury (AKI) remains a significant complication following cardiac surgery, associated with increased morbidity and mortality. The early detection of AKI is limited by the cost, availability, and unclear clinical utility of the current biomarkers. This study aimed to evaluate the red cell distribution width (RDW) on ICU admission as a predictor of postoperative AKI. **Methods**: We conducted a retrospective analysis of adult patients undergoing isolated coronary artery bypass grafting (CABG) or combined CABG and aortic valve surgery at a tertiary cardiac surgery centre (University Hospital of Siena, Italy) between January 2015 and December 2020. AKI was defined according to the KDIGO criteria. The RDW was measured preoperatively (T0), at ICU admission (T1), and at 24 (T2) and 48 h (T3) postoperatively. Temporal RDW changes (ΔRDW) were also calculated. Multivariate logistic regression identified independent predictors of AKI, and receiver operating characteristic (ROC) analysis evaluated the predictive accuracy. **Results**: A total of 456 patients were included, with an overall AKI incidence of 31%. Patients developing AKI exhibited significantly higher RDW at all measured time points, especially at ICU admission. Multivariate analysis identified age, RDW (OR 1.19, 95% CI: 1.03–1.37, *p* = 0.016) and serum creatinine at ICU admission, and elevated lactate at T2 as independent AKI predictors. In subgroup analyses, RDW at ICU admission remained significantly associated with AKI in patients who were not transfused, but not in patients who were. **Conclusions**: In this study, a high RDW at ICU admission represented an early postoperative marker independently associated with AKI after cardiac surgery, particularly in patients who did not receive transfusion.

## 1. Introduction

Acute kidney injury (AKI) is a frequent complication in patients undergoing cardiovascular surgery, occurring in up to 42% of cases, depending on the type of surgery and the definition of AKI employed [1]. The early prediction or diagnosis of AKI following cardiac surgery is clinically critical, as postoperative AKI is associated with adverse short-term outcomes and significantly increased long-term morbidity and mortality [2]. The diagnosis of AKI based on serum creatinine elevation is often delayed, as detectable increases can take several days to manifest. Thus, developing accurate predictive models to facilitate earlier and more effective management of AKI is crucial. Although several biomarkers have been proposed for the early risk stratification and diagnosis of AKI, their widespread clinical adoption has been limited by factors such as reagent cost, limited availability, and unclear effects on clinical decision-making. Conversely, red blood cell distribution width (RDW), a measure of erythrocyte size variability (anisocytosis), is routinely available without additional cost or analytical complexity. Recently, RDW has gained attention as a potential surrogate biomarker given its correlations with several cardiovascular conditions. Elevated RDW levels have been associated with coronary artery disease, myocardial infarction risk, recurrent coronary events, restenosis after stenting, myocardial scar burden, mortality, and adverse cardiovascular outcomes [3]. Furthermore, increased RDW values correlate with poorer prognosis in patients with heart failure, myocardial infarction, and coronary artery disease [4,5] and serve as independent predictors of both in-hospital mortality and long-term survival following isolated coronary artery bypass grafting (CABG) surgery [6]. While recent studies have recognised the prognostic role of RDW in cardiac surgery, there is limited and heterogeneous evidence regarding its specific value for predicting AKI [7,8,9]. An elevated RDW has been associated with systemic inflammation, oxidative stress, and reduced erythrocyte deformability, factors that may impair microvascular oxygen delivery [10,11]. Since renal hypoxia and inflammatory activation are central in the pathogenesis of postoperative AKI [1], RDW may represent an integrated marker of susceptibility to kidney injury in cardiovascular surgery. Given the high incidence and substantial impact of AKI in patients undergoing cardiac surgery, the present study aims to evaluate whether RDW can be employed as a simple, inexpensive, and effective biomarker to predict postoperative AKI in patients undergoing cardiac surgery. Further, due to the complex interrelationship between RDW, AKI, and red blood cell transfusion, we seek to investigate whether the predictive value of RDW for AKI differs between patients receiving and not receiving transfusion [7,8].

## 2. Materials and Methods

### 2.1. Study Population

We conducted a retrospective observational study, analysing data from patients undergoing isolated CABG or combined CABG and aortic valve surgery at the Cardiothoracic and Vascular Department of the University Hospital of Siena, Italy, between January 2015 and December 2020. The study protocol was approved by the local Ethics Committee (protocol number 22046, dated 11 April 2022), and written informed consent was obtained from all enrolled patients. Exclusion criteria included age less than 18, off-pump CABG, and chronic kidney failure according to the Kidney Disease Improving Global Outcomes (KDIGO) [12,13].

### 2.2. Data Collection

Blood samples for hematologic parameter determination and blood gas analysis (BGA) were collected at predefined intervals: the day before surgery (T0), at ICU admission immediately after surgery (T1), and then 24 h (T2) and 48 h (T3) postoperatively. T0 represented the preoperative baseline RDW value, while T1 captured the immediate postoperative state. This approach enabled assessment of both pre-existing vulnerability and early perioperative changes potentially related to cardiopulmonary bypass, inflammatory activation, and transfusion. BGA was performed using a dedicated analyser (GEM Premier™ 4000, Werfen, Le Pré-Saint-Gervais, France). Complete blood counts, including RDW, were analysed using a Sysmex K-1000 auto-analyser (Block Scientific, Bohemia, NY, USA). The normal laboratory range for RDW at our centre was 10.9–14.5%. To assess the temporal variation in RDW, we calculated ΔRDW values, defined as differences between RDW measurements obtained at the consecutive time points (T1-T0, T2-T1, T2-T0, and T3-T2).

### 2.3. Anaesthesia Cardiopulmonary Bypass, and Myocardial Protection

Patients underwent standardised cardiothoracic anaesthesia according to institutional protocols. Induction and maintenance were performed using intravenous and/or inhalational agents at the discretion of the attending anaesthesiologist. Hemodynamic management and cardiopulmonary bypass strategies followed routine clinical practice. Anaesthetic agents are not known to acutely affect RDW values, which primarily reflect erythrocyte size variability and erythropoietic dynamics.

### 2.4. Study Outcomes

The primary outcome of this study was to evaluate whether RDW can predict the occurrence of AKI; AKI was defined according to the KDIGO criteria as an increase in sCr by ≥0.3 mg/dL (≥26.5 μmol/L) within 48 h or an increase in sCr to ≥1.5 times baseline [10]. Patients discharged within 24 or 48 h of admission were followed up during their hospital stay to ensure they did not develop AKI after discharge from the ICU. The secondary outcome was to evaluate the ability of RDW to identify the onset of AKI changes between patients who did and did not receive transfusion.

### 2.5. Statistical Analysis

Statistical analyses were performed using the Statistical Package for Social Sciences (SPSS; version 25, IBM Corp., Armonk, NY, USA). Normality of data distribution was assessed using the Kolmogorov–Smirnov test. Continuous variables are expressed as mean ± standard deviation (SD), and comparisons were made using Student’s *t*-test for normally distributed variables or Mann–Whitney U-test for non-normally distributed variables. Categorical variables are presented as absolute number and percentages (%) and were compared using the chi-square test or Fisher’s exact test, as appropriate. To identify variables independently associated with postoperative AKI, multivariate logistic regression analysis was performed using AKI occurrence in the ICU as the dependent variable. Type of surgery (e.g., isolated CABG, combined CABG and valve surgery) was evaluated in univariate analysis and included as a covariate in multivariable logistic regression models assessing predictors of AKI. To assess whether the association between RDW and AKI differed according to transfusion status, an interaction term between RDW at ICU admission (T1) and PRBC transfusion status (RDW × transfusion) was also included in the multivariable logistic regression model.

Before inclusion in the multivariate model, potential collinearity among variables was assessed and excluded. Variables with a *p*-value < 0.2 in the univariate analysis were included in the multivariate regression. Clinically relevant variables previously associated with cardiac-surgery-associated AKI, including cardiopulmonary bypass duration and aortic cross-clamp time, were evaluated in univariate analyses and considered during the stepwise construction of the multivariable logistic regression models. Results are reported as odds ratios (ORs) with 95% confidence intervals (CIs). Model calibration and goodness-of-fit were assessed using the Hosmer–Lemeshow test. The discriminatory capability of RDW in predicting AKI was further evaluated using receiver operating characteristic (ROC) curves. The area under the ROC curve (AUC) was calculated together with the corresponding sensitivity and specificity values. A *p*-value < 0.05 was considered statistically significant. Based on a power analysis using a two-tailed *t*-test, a minimum sample size of 424 patients was required to achieve 95% power with an effect size of 0.5 and an alpha level of 0.05. Accounting for a potential 5% dropout rate, a total sample size of at least 450 patients was considered necessary.

## 3. Results

### 3.1. Baseline Characteristics

**Figure 1:**
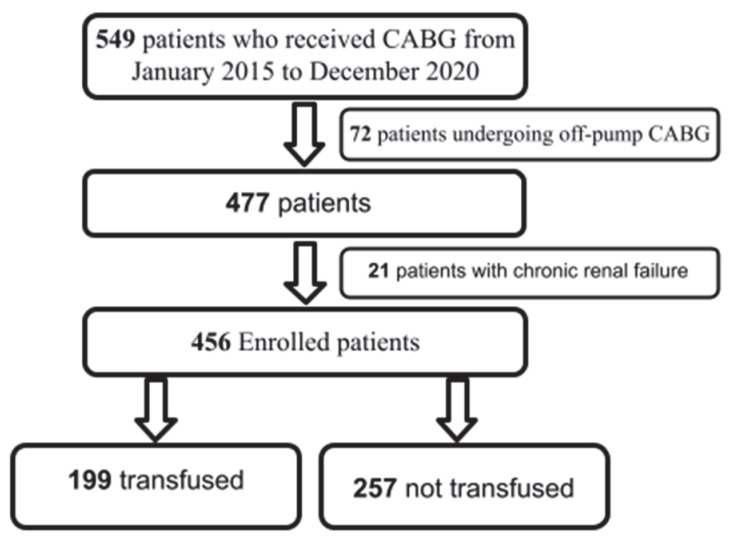
Study sample selection flow diagram.

From a total of 549 patients, 72 were excluded due to off-pump CABG and 21 because of chronic renal failure; thus, 456 patients were included in the final analysis (Figure 1). Among these, 361 patients underwent isolated CABG (79%), while 95 (21%) had combined CABG and aortic valve surgery. None of the patients received packed red cell transfusions intraoperatively. The mean age was 70 ± 9 years, and 349 patients were male (76%) (Table 1).

### 3.2. Acute Kidney Injury

The overall AKI incidence was 31% (n = 143); AKI was diagnosed in all cases within 48 h of admission to the ICU; after discharge from the ICU, none of the patients developed AKI. Continuous renal replacement therapy (CRRT) was required in only 2 out of 143 patients (1%). Patients developing AKI were significantly older and more frequently presented than others with chronic atrial fibrillation, arterial hypertension, and previous cardiac surgery (Table 1). Type of surgery (isolated CABG vs. combined CABG and valve surgery) was not significantly associated with postoperative AKI (*p* = 0.19). CPB duration was significantly longer in the AKI group; lactate levels were significantly elevated in patients with AKI at all postoperative time points. Patients with AKI also had significantly higher SAPS II scores, more respiratory failure, infectious complications, longer ICU stays, and higher mortality than patients without AKI (Table 2).

### 3.3. RDW Values

**Figure 2:**
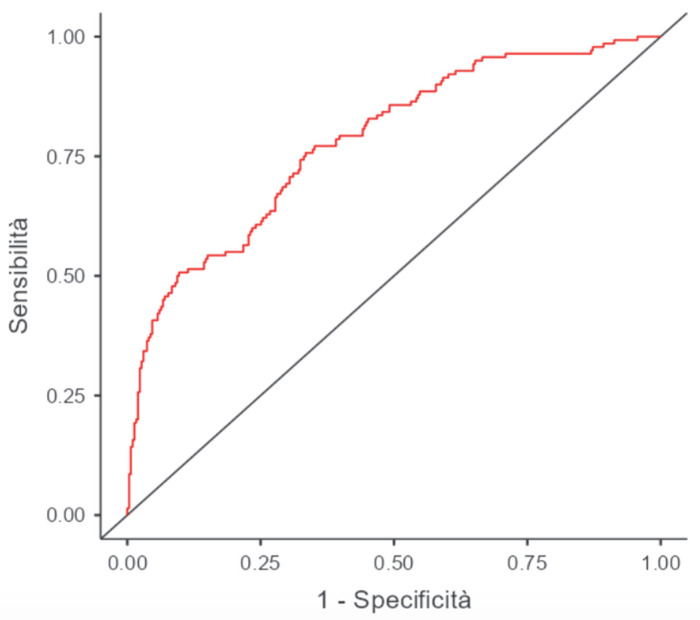
ROC curve of the model in patients who did and did not receive transfusion (AUC 0.782).

RDW values were significantly higher in patients who developed AKI at all time points (Figure 2) than in others. The ΔRDW values between admission and preoperative values, as well as for other time points, were also significantly higher in patients with AKI compared to patients without AKI.

RDW values at all predefined time points in patients with and without AKI are illustrated in Figure 2.

### 3.4. Predictors of AKI

**Figure 3:**
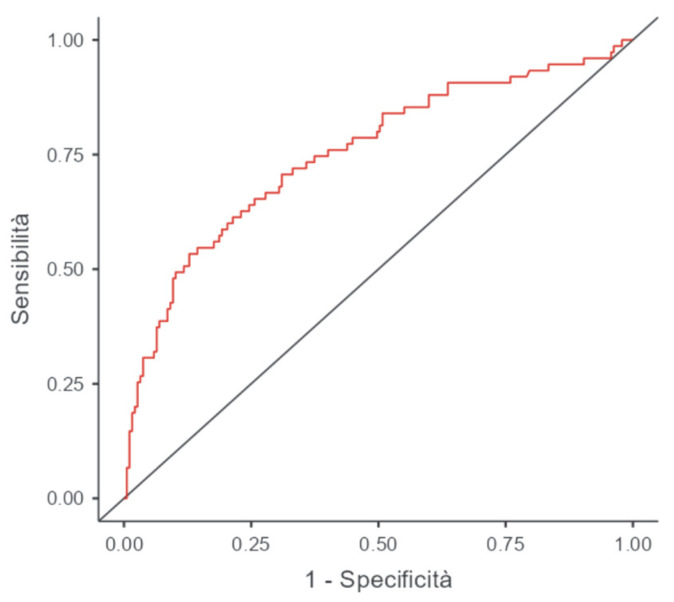
ROC curve model of patients who were not transfused (AUC 0.75).

Multivariate logistic regression analysis identified RDW at ICU admission (OR 1.19; 95% CI: 1.03–1.37, *p* = 0.016), together with elevated lactate at T2, age, and serum creatinine at ICU admission, as independent predictors of AKI (Table 3). In an additional analysis si mi smincluding an interaction term between RDW and PRBC transfusion (RDW × transfusion), no significant interaction was observed (OR 1.05, 95% CI 0.71–1.50, *p* = 0.8), suggesting that the association between RDW and AKI did not significantly differ with transfusion status. ROC analysis indicated an optimal RDW cutoff at ICU admission of 14.1% for AKI prediction (sensitivity 46%; specificity 75%; AUC of 0.782). The ROC curve for AKI prediction based on RDW at ICU admission is shown in Figure 3.

### 3.5. Secondary Outcome

Among 257 patients who were not transfused (81% isolated CABG, 19% combined surgery), the incidence of AKI was 28%. Baseline characteristics and comorbidities of these patients who did not receive transfusion are reported in Supplementary Table S1. Patients who developed AKI were significantly older, had longer CPB times, and had higher lactate levels from ICU admission through postoperative day 2. They also had higher SAPS II scores, more respiratory and infectious complications, higher tracheostomy rates, and prolonged ICU stays. Perioperative and early ICU variables in patients without transfusion are summarised in Supplementary Table S2. The RDW values at ICU admission and during the first two postoperative days were significantly higher in patients with AKI, while the preoperative RDW did not differ between groups. ΔRDW from baseline was consistently greater in patients with AKI. In the multivariate analysis, RDW at ICU admission emerged as an early postoperative marker independently associated with AKI (OR 1.29, CI: 1.02–1.62; *p* = 0.032). ROC analysis identified an RDW cutoff of 14.1% at ICU admission to predict AKI (AUC of 0.750; sensitivity 20%, specificity 68%). The results of the multivariate logistic regression analysis in patients without transfusion are presented in Supplementary Table S3.

Among the 199 patients who received transfusion, AKI occurred in 36%. The baseline characteristics and comorbidities of these patients are reported in Supplementary Table S4. Differences between patients with and without AKI were similar to those in patients receiving transfusions. RDW values were significantly higher in patients with AKI at all time points, including preoperative measurements; however, ΔRDW values did not differ between groups. Perioperative and early ICU variables in patients receiving transfusion are summarised in Supplementary Table S5. In the multivariate analysis, RDW was not an independent predictor of AKI. The results of the multivariate logistic regression analysis in patients receiving transfusion are presented in Supplementary Table S6.

## 4. Discussion

In this study, we found that elevated RDW along with serum creatinine (sCr), both measured at ICU admission; peak lactate on the first postoperative day; and advanced age were independently associated with the development of AKI after cardiac surgery. Because AKI was defined within 48 h after surgery, the RDW measured at ICU admission should be interpreted as an early postoperative marker rather than a purely preoperative predictor. Its independent association after adjustment for serum creatinine at the same time point suggests that RDW captures additional pathophysiological mechanisms, such as inflammatory activation and microvascular dysfunction, beyond early renal functional changes. Although baseline renal function is an important determinant of postoperative AKI risk and was captured by baseline serum creatinine (T0), serum creatinine measured at ICU admission (T1) was included in the multivariable model as it represents the earliest postoperative indicator of renal function and integrates both baseline renal vulnerability and early perioperative renal stress. This result was confirmed in a subgroup analysis including patients not receiving transfusion. This finding is consistent with previous studies showing that transfusion can confound RDW values, potentially masking its prognostic role in postoperative outcomes [13].

Few prior studies have examined the relationship between an elevated RDW and the development of AKI after cardiac surgery. In Zou et al.’s study, RDW was not an independent predictor of AKI [7]. Conversely, other investigations reported an association between higher RDW values and an increased risk of postoperative AKI requiring renal replacement therapy, as well as higher mortality among patients undergoing valve surgery [9]. Our findings confirm and extend these observations by demonstrating that RDW measured at ICU admission independently predicts the risk of AKI specifically in patients who did not receive transfusion during cardiac surgery. Given that previous studies did not adequately report or adjust for perioperative red blood cell transfusion, this confounding factor might have contributed to the inconsistency of our results with earlier results.

The mechanisms underlying the association between elevated RDW and AKI remain incompletely understood. RDW, which reflects erythrocyte size variability, may increase in response to systemic inflammation, oxidative stress, impaired erythropoiesis, or nutritional deficiencies—conditions frequently encountered in patients undergoing cardiac surgery [3,14,15]. These factors can impair microvascular blood flow, increase blood viscosity, and promote tissue hypoxia, thereby heightening susceptibility to AKI [14]. Further, reduced erythrocyte deformability associated with higher RDW may worsen renal microcirculatory perfusion, contributing directly to organ dysfunction [10]. A key unresolved question is whether anisocytosis is merely a surrogate marker of underlying pathological processes or whether it plays a causal role in organ injury, including AKI. The current evidence suggests that both mechanisms are plausible: chronic inflammation and metabolic stress may drive anisocytosis, while anisocytosis may aggravate microcirculatory disturbances and amplify ischaemic injury in vulnerable organs such as the kidneys [11,14].

Consistent with the prior literature, our results confirm serum creatinine as a fundamental biomarker for assessing perioperative renal function and stratifying risk in cardiac surgery populations. Even small increases in creatinine that do not meet standard AKI thresholds are associated with worsened outcomes [16,17]. Our findings also reinforce the existing evidence demonstrating that high lactate levels are strongly linked to postoperative morbidity and mortality. Elevated lactate, a potential indicator of tissue hypoperfusion, reliably predicts adverse outcomes, including AKI, after cardiac surgery [18,19,20]. Taken together, our findings have meaningful clinical implications. Routine RDW measurement at ICU admission may enhance perioperative risk stratification, particularly in patients who do not receive transfusions in cardiac surgery. Identifying individuals at elevated risk of AKI using a simple, inexpensive, and widely available biomarker such as RDW could help clinicians tailor preventive strategies, including personalised fluid management, early haemodynamic optimisation, and judicious avoidance of nephrotoxic agents. Moreover, older adult patients and those with pre-existing renal impairment or chronic inflammatory conditions may derive particular benefit from RDW monitoring, given their heightened vulnerability to postoperative complications such as AKI. Taken together, these findings suggest that RDW represents a simple and readily available biomarker to improve early postoperative risk stratification for AKI after cardiac surgery, although further prospective studies are required to validate its role in clinical decision-making.

Our study has several limitations. First, its retrospective, single-centre design restricts the generalisability of the findings, underscoring the need for confirmation in prospective, multicentre cohorts. Nonetheless, the relatively large sample size and the standardised perioperative and ICU protocols used at our institution partially mitigate this limitation. Second, the variability in RDW thresholds reported across previous studies highlights the need for a standardised cut-off to improve comparability and reproducibility. In our analysis, ROC-derived optimal thresholds were applied, enhancing the clinical relevance within the studied population. Although we performed rigorous multivariate adjustments, residual confounding from unmeasured variables, such as postoperative bleeding, or variables not retained in the final model cannot be completely excluded given the retrospective observational design of this study. However, we incorporated several well-established AKI risk factors into the models, reducing the likelihood of significant unaccounted confounding. Another limitation is that we did not systematically assess preoperative medications (e.g., statins, ACE inhibitors, beta-blockers) or other perioperative management strategies that may influence AKI development. Nevertheless, perioperative care was broadly standardised according to institutional guidelines, which should limit the impact of this omission. Finally, the lack of data on long-term renal function and extended clinical outcomes prevented the assessment of the broader prognostic implications of elevated RDWs after cardiac surgery. Despite this limitation, our findings provide important early postoperative insights and form a basis for future research. Prospective studies integrating these additional parameters are required to evaluate the prognostic utility of RDW more comprehensively and to refine clinical risk prediction models for cardiac-surgery-associated AKI.

## 5. Conclusions

In this study, a high RDW at ICU admission was an early postoperative marker independently associated with AKI following cardiac surgery. However, it appeared particularly robust in patients who did not receive red blood cell transfusions.

## Figures and Tables

**Table 1:** Comparison of demographic data and comorbidities in patients and in AKI and no AKI groups. Data are presented as mean ± standard deviation or number (%), as appropriate.

	Patients N = 456	AKI Group N = 143 (31%)	No AKI Group N = 313 (69%)	*p*-Value
**Age**, years	70 ± 9	72 ± 9	68 ± 9	<0.001
**Male sex**, N (%)	349 (76)	109 (76)	240 (76)	0.916
**CAF**, N (%)	19 (4)	12 (8)	7 (2)	0.002
**PAF**, N (%)	16 (3)	7 (5)	9 (3)	0.277
**Previous myocardial infarction** N (%)	144 (31)	42 (29)	102 (33)	0.493
**Previous stroke**, N (%)	11 (2)	6 (4)	5 (2)	0.093
**Hypertension**, N (%)	379 (83)	128 (89)	251 (80)	0.010
**Peripheral vascular disease**, N (%)	59 (13)	20 (14)	39 (12)	0.652
**Diabetes mellitus**, N (%)	157 (34)	53 (37)	104 (33)	0.437
**Thyroid disease**, N (%)	28 (6)	11 (8)	17 (5)	0.351
**Dyslipidaemia**, N (%)	305 (67)	98 (68)	207 (66)	0.614
**Asthma**, N (%)	8 (2)	2 (1)	6 (2)	0.690
**COPD**, N (%)	49 (11)	19 (13)	30 (10)	0.241
**Cancer history**, N (%)	42 (9)	17 (12)	25 (8)	0.181
**Previous cardiac surgery**, N (%)	23 (5)	13(9)	10 (3)	0.008
**CABG**, N (%)	361 (79)	108 (30)	253 (70)	0.19
**Combined CABG**	95 (21)	35 (37)	60 (63)	0.19

CAF: chronic atrial fibrillation; PAF: paroxysmal atrial fibrillation; COPD: chronic obstructive pulmonary disease; CABG: coronary artery bypass grafting.

**Table 2:** Comparison of preoperative, intraoperative and variables related to the first 48 h after admission in the ICU.

	Patients N = 456	AKI Group N = 143 (31%)	No AKI Group N = 313 (69%)	*p*-Value
**CPB time**, min	123 ± 40	131 ± 40	119 ± 40	0.002
**Aortic-cross clamp time**, min	92 ± 33	96 ± 35	90 ± 32	0.066
**Peak lactate CPB, mmol/L**	2.9 ± 1.2	3.2 ± 1.6	2.8 ± 0.9	0.031
**Peak lactate T1 mmol/L**	2.4 ± 1.9	3.2 ± 2.8	2.1 ± 1.4	<0.001
**Peak lactate T2 mmol/L**	2.6 ± 1.6	3.3 ± 2.1	2.3 ± 1.2	<0.001
**Peak lactate T3 mmol/L**	1.5 ± 1.0	1.9 ± 1.4	1.3 ± 0.4	<0.001
**T0 sCr**, mg/dL	1.0 ± 0.3	1.0 ± 0.3	0.9 ± 0.2	0.126
**T1 sCr**, mg/dL	0.9 ± 0.3	1.1 ± 0.3	0.8 ± 0.2	<0.001
**T2 sCr**, mg/dL	1.1 ± 0.4	1.4 ± 0.4	0.9 ± 0.2	<0.001
**T3 sCr**, mg/dL	1.2 ± 0.6	1.6 ± 0.7	0.9 ± 0.2	<0.001
**T0 RDW**, %	13.9 ± 1.5	14.2 ± 1.8	13.8 ± 1.4	0.007
**T1 RDW**, %	13.9 ± 1.7	14.4 ± 2.0	13.7 ± 1.4	<0.001
**T2 RDW**, %	14.3 ± 1.6	14.8 ± 2.0	14.1 ± 1.4	<0.001
**T3 RDW**, %	14.7 ± 1.8	15.1 ± 1.9	14.4 ± 1.7	<0.001
**ΔRDW T1-T0**, %	0.08 ± 1.3	0.38 ± 1.93	−0.06 ± 0.91	<0.001
**PRBC transfusion, n (%)**	199 (44)	72 (36%)	127 (64%)	0.051
**SAPSII**	32 ± 7	35 ± 7	30 ± 6	<0.001
**Length of stay in ICU**, days	3 ± 5	6 ± 9	2 ± 2	<0.001
**ICU mortality**, N (%)	9 (2)	8 (6)	1 (0.3)	<0.001

CPB: cardiopulmonary bypass; sCr: serum creatinine; RDW: red cell distribution width; PRBC, packed red blood cell; SAPSII: Simplified Acute Physiology Score II; ICU: intensive care unit.

**Table 3:** Multivariable logistic regression analyses for AKI: comparison between full model (“enter”) and stepwise-selected model. Odds ratios (ORs) with 95% confidence intervals (CIs) are reported. The “enter” model included all candidate variables, while the stepwise model included only variables retained after stepwise selection based on univariate screening.

	Enter			Stepwise		
	OR	95% Confidence Interval	*p*-Value	OR	95% Confidence Interval	*p*-Value
Age	1.02	0.97–1.07	0.32	1.04	1.01–1.06	0.006
CAF	2.27	0.49–10.55	0.29	--	--	--
Hypertension	0.75	0.52–5.90	0.18	--	--	--
Previous Cardiac Surgery	0.58	0.24–1.38	0.22	--	--	--
CPB time	1.00	0.98–1.02	0.38	--	--	--
Peak lactate CPB	1.26	0.92–1.74	0.91	--	--	--
Peak lactate T1	1.05	0.81–1.34	0.45			
Peak lactate T2	1.28	1.01–1.52	0.04	1.35	1.16–1.57	<0.001
Peak lactate T3	1.47	0.82–2.66	0.14	--	--	--
T1 sCr	0.35	0.05–2.21	0.17	17.51	7.22–42.44	<0.001
T2 sCr	1.72	0.27–10.97	0.83	--	--	--
T3 sCr	1.08	0.43–2.71	0.06	--	--	--
T0 RDW	0.97	0.73–1.30	0.77	--	--	--
T1 RDW	0.71	0.33–1.56	0.42	1.19	1.03–1.37	0.016
T2 RDW	1.27	0.52–3.09	0.47	--	--	--
T3 RDW	1.03	0.54–1.95	0.92	--	--	--
PRBC Transfusion	1.38	0.69–2.73	0.28	--	--	--
SAPSII	1.06	0.99–1.13	0.14	--	--	--

AKI: acute kidney injury; CAF: chronic atrial fibrillation; CPB: cardiopulmonary bypass; sCr: serum creatinine; RDW: red cell distribution width; PRBC: packed red blood cell; SAPSII: Simplified Acute Physiology Score II; T0: preoperative measurement; T1: ICU admission; T2–T3, subsequent postoperative time points.

## Data Availability

The data presented in this study are available on request from the corresponding author due to privacy and ethical restrictions.

## Supplementary Materials

The following supporting information can be downloaded at: https://www.mdpi.com/article/10.3390/jcm15062403/s1, Table S1: Baseline demographic characteristics and comorbidities in patients who did not receive PRBC transfusion. Data are presented as mean ± SD or number (%), as appropriate; Table S2: Perioperative and early postoperative variables in non-transfused patients; Table S3: Independent predictors of AKI in non-transfused patients; Table S4: Baseline characteristics in patients who received PRBC transfusion; Table S5: Perioperative and early postoperative variables in transfused patients; Table S6: Independent predictors of AKI in transfused patients.
