# Supplementary material for: Prognostic Value of Red Blood Cell Distribution Width in Predicting Acute Kidney Injury After Cardiac Surgery: A Retrospective Cohort Study
Source: J Clin Med. 2026 Mar 21;15(6):2403. doi: 10.3390/jcm15062403 (PMC13027086; doi:10.3390/jcm15062403)
Supplement: Supplementary file 1 [file jcm-15-02403-s001.zip › jcm-4148559-supplementary.pdf]

Supplementary Tables

Supplementary Table S1. Baseline demographic characteristics and comorbidities in patients who did not receive PRBC transfusion. Data are presented as mean ± SD or number (%), as appropriate.

Variable	Overall (N = 257)	AKI (N = 71, 28%)	No AKI (N = 186, 72%)	p-value
**Age, years**	68 ± 9	70 ± 9	67 ± 9	0.001
**Male sex, n (%)**	218 (85)	58 (81)	160 (86)	0.387
**Chronic AF, n (%)**	13 (5)	8 (11)	5 (3)	0.005
**Hypertension, n (%)**	201 (78)	61 (86)	140 (75)	0.074
**Diabetes mellitus, n (%)**	87 (34)	28 (39)	59 (32)	0.242
**Neoplasia, n (%)**	25 (10)	12 (17)	13 (7)	0.016

Supplementary Table S2. Perioperative and early postoperative variables in non-transfused patients.

Variable	AKI	No AKI	p-value
**CPB time**	132 ± 38	112 ± 35	<0.001
**Peak lactate T2**	3.1 ± 2.2	2.2 ± 1.1	<0.001
**T1 RDW (%)**	13.9 ± 1.7	13.4 ± 1.2	0.008
**ΔRDW T1–T0**	0.02 ± 1.51	-0.20 ± 0.95	0.001
**SAPS II**	34 ± 7	30 ± 6	<0.001
**ICU length of stay**	4 ± 7	2 ± 1	<0.001

Supplementary Table S3. Independent predictors of AKI in non-transfused patients.

Variable	OR	95% CI	p-value
**CPB time**	1.01	1.00–1.02	0.011
**Peak lactate T2**	1.35	1.11–1.63	0.002
**RDW at T1**	1.29	1.02–1.62	0.032

Supplementary Table S4. Baseline characteristics in patients who received PRBC transfusion.

Variable	Overall (N = 199)	AKI (N = 72, 36%)	No AKI (N = 127, 64%)	p-value
**Age, years**	72 ± 9	74 ± 8	71 ± 9	0.008
**Male sex, n (%)**	131 (66)	51	80	0.262
**Diabetes mellitus, n (%)**	70 (35)	25	45	0.888

Variable	Overall (N = 199)	AKI (N = 72, 36%)	No AKI (N = 127, 64%)	p-value
**Hypertension, n (%)**	178 (90)	67	111	0.119

Supplementary Table S5. Perioperative and early postoperative variables in transfused patients.

Variable	AKI	No AKI	p-value
**Peak lactate T2**	3.4 ± 1.9	2.4 ± 1.4	<0.001
**T1 RDW (%)**	14.8 ± 2.2	14.1 ± 1.6	<0.001
**ΔRDW T1–T0**	0.7 ± 2.2	0.14 ± 0.81	0.062
**SAPS II**	36 ± 6	31 ± 6	<0.001
**ICU mortality**	7 (10%)	1 (1%)	0.002

Supplementary Table S6. Independent predictors of AKI in transfused patients.

Variable	OR	95% CI	p-value
**SOFA**	1.61	1.22–2.14	<0.001
**Fluid balance >1000 ml at 24h**	3.31	1.27–8.58	0.014
**Peak lactate T2**	1.43	1.09–1.86	0.009
